# Maternal stress and depressive symptoms and adolescents’ body mass index: a prospective study

**DOI:** 10.1186/s12889-021-10721-z

**Published:** 2021-04-07

**Authors:** Maaike Koning, Jacqueline Vink, Tommy L. S. Visscher, Junilla Larsen

**Affiliations:** 1 Developmental Psychology, Behavioural Science Institute, Radboud University, P.O. Box 9104, 6500 HE Nijmegen, The Netherlands; 2 Department of Healthy Society, Knowledge Centre for Health and Social work, Windesheim University of Applied Sciences, Zwolle, The Netherlands; 3 Hanze University of Applied Sciences, Groningen, The Netherlands

**Keywords:** Longitudinal study, Adolescence, Overweight, Maternal stress, Maternal depression, BMI

## Abstract

**Background:**

Growing evidence suggests that maternal mental health issues are associated with (young) children’s weight outcomes. However, most studies have been limited by cross-sectional designs and have been aimed at (younger) children. The current prospective study focuses on the link between maternal mental health (i.e., psychological stress and depressive symptoms) and adolescents’ zBMI development.

**Methods:**

The participants in the present study were part of wave 1 and 2 of a longitudinal study on Dutch adolescents’ and their parents’ health behavior. Adolescents (aged 10–14) and their parents were recruited through six secondary schools in the South and the East of the Netherlands. For this study, we only included biological mothers and their adolescent children who participated in both waves, with data on the main measures in both waves, leaving a final sample of 336 biological mother-child dyads. Adolescents height and weight were measured, and both parents and adolescents filled in validated questionnaires on perceived stress and depressive symptoms and answered additional questions concerning domain-specific stress. Regression analyses were performed in R to examine longitudinal links between maternal stress and depressive symptoms at baseline (T1) and adolescents’ BMI standard deviation scores (zBMI) 6 months later (T2), corrected for baseline zBMI and covariates.

**Results:**

Maternal general perceived stress (β = .20, *p* = .002) at T1 preceded higher adolescents’ zBMI at T2, after controlling for baseline zBMI and other covariates, whereas maternal depressive symptoms at T1 (β = −.05, *p* = .44) and other domain-specific stress did not (maternal financial stress, maternal stress at work, maternal stress at home). Additionally, lower educational level among adolescents (β = .16, *p* = .001) and adolescent depressive symptoms (β = .16, *p* = .001) was associated with a higher zBMI at T2.

**Conclusions:**

Results suggest that maternal general stress, but not depressive symptoms, may influence adolescents’ weight development. Our findings warrant future investigation on whether and how general stress among mothers may predict weight increases of their adolescent offspring.

## Background

Adolescence is a vulnerable period for the development of overweight, a pressing public health issue [1–8], and is characterized by critical changes in body composition, physical activity and eating behaviors, and psychological adjustments [5, 9, 10]. Being overweight and especially being obese as an adolescent increases the risk of becoming an overweight adult [11–15], and has been associated with serious comorbidities in adulthood [16, 17]. While overweight and obesity have multifactorial causes, parents have a substantial influence on the development of children’s and adolescents’ eating behaviors [18, 19], food consumption [20–22], and weight gain trajectories [23, 24]. The role of mothers is especially important because in most households nowadays, mothers are still the most important caregivers [25], and manage most of the day-to-day child-care tasks [26]. They are considered to be the primary gatekeeper of the home food environment [27]. To date, growing evidence suggests that maternal mental health issues (i.e., psychological stress and depressive symptoms) are associated with increased risk rates of weight gain, obesity and related weight-related behaviors (i.e., eating, physical activity and sleep patterns) in children and adolescents [28–38]. However, most studies have been limited by cross-sectional designs and have been aimed at (younger) children. The current prospective study focuses on the link between maternal mental health and adolescents’ weight development.

It has been hypothesized that maternal stress and depressive symptoms may influence their child’s weight related behaviors through three primary pathways [39]. First, maternal stress may alter mothers’ own physical activity, sedentary behavior and dietary intake, which may impact children’s and adolescents’ behaviors through modelling and household exposure (e.g., less healthy family meals) [40]. Additionally it is known that depressed mothers have difficulty providing healthful food choices [41–43], and modeling physical activity behaviors relative to non-depressed mothers [44]. There is evidence that changes in maternal feeding styles and patterns due to stress and depression can have a significant impact on children’s food composition and energy intake by for example preparing convenient but unhealthy meals to help manage time [25, 45]. Second, maternal stress and depression may affect parenting behaviors or mother child interactions. Mothers experiencing high levels of stress may spend less time with their children [46] and may be less responsive in their interaction with their children [47]. Maternal depressive symptoms, such as negative affect and inactivity, can influence child weight related behaviors by directly affecting parenting behaviors, reducing maternal sensitivity to and nurturance of the child’s needs [37, 48] and by facilitating less positive parent-child interactions and less family cohesion [49–51]. Third, maternal stress and depression can directly influence children’s behavior through alterations in the stress response of the child itself [40], responding to maternal stress with an increased biological or psychological stress response .

These pathways explaining the link between maternal mental health and child weight development may particularly be important during adolescence. That is, parents continue to play a decisive role in this age period, for instance through weight-related parenting and specific rules that are associated with greater adolescent nutrition knowledge [52]. This nutrition knowledge may, in turn, prevent unhealthy choices during adolescence when children become more autonomous [52] and are increasingly exposed to more diverse (unhealthier) food environments. Thus, what parents do, both in terms of their own behaviors as well as their food parenting, may prepare adolescents to deal with these more diverse unhealthy food environments, preventing excessive food intake and subsequent weight gain. Moreover, the third pathway explaining the link between maternal mental health and child weight development through child mental health issues may also be particularly important during adolescence, when depression and stress symptoms are more prevalent compared to childhood [53].

To date, previous studies have found that maternal general stress was consistently associated with greater risk for childhood overweight and obesity [28], while the association between depressive symptoms and child overweight was found to be more inconsistent [54], varying by gender and age [55] and measure of depression [54]. Importantly, the majority of studies so far have focused on maternal stress or maternal depression or depressive symptoms exclusively, while longitudinal assessment of both maternal stress and depressive symptoms in relation to childhood and adolescents’ BMI are rare. Our study is unique in examining the longitudinal link of both stress and depressive symptoms with adolescents’ zBMI development.

Stress may seem to represent one generalizable phenomenon, but there are many sub-classifications of stress, including general perceived stress and perceived stress limited to a specific domain (e.g., financial, work-related or the home environment) [34]. Each of these different types of stress experienced by mothers may differentially relate to their children’s weight-related behaviors and subsequent weight development. For example, financial stress may particularly lead parents to choose low-cost energy-dense food alternatives because of financial constraints [56, 57] and has been found to be positively associated with children’s weight status [30]. Moreover, higher work-related stress may also particularly result in easy energy-dense food alternative because of time constraints. To date, work-related stress among parents of adolescents has been associated with less healthful family food environment characteristics, including less frequent family meals, more frequent fast food for family meals, and less time spent on food preparation [58].

The main aim of the current prospective study is to examine the link between diverse forms of stress, general and domain-specific, and depressive symptoms with adolescents’ weight outcomes. Our general hypothesis is that maternal stress and depressive symptoms are linked with increases in adolescents’ zBMI over time. Characteristics like educational level of the mother and of the adolescent, adolescents’ gender, maternal BMI, maternal marital status, adolescent stress and depressive symptoms, as also the quality of the parent-child relationship rated by parents, will be considered as potential confounding factors for the association of maternal stress and depressive symptoms with adolescent weight outcomes as these are associated with BMI in adolescents [59–70].

## Methods

### Participants

**Fig. 1:**
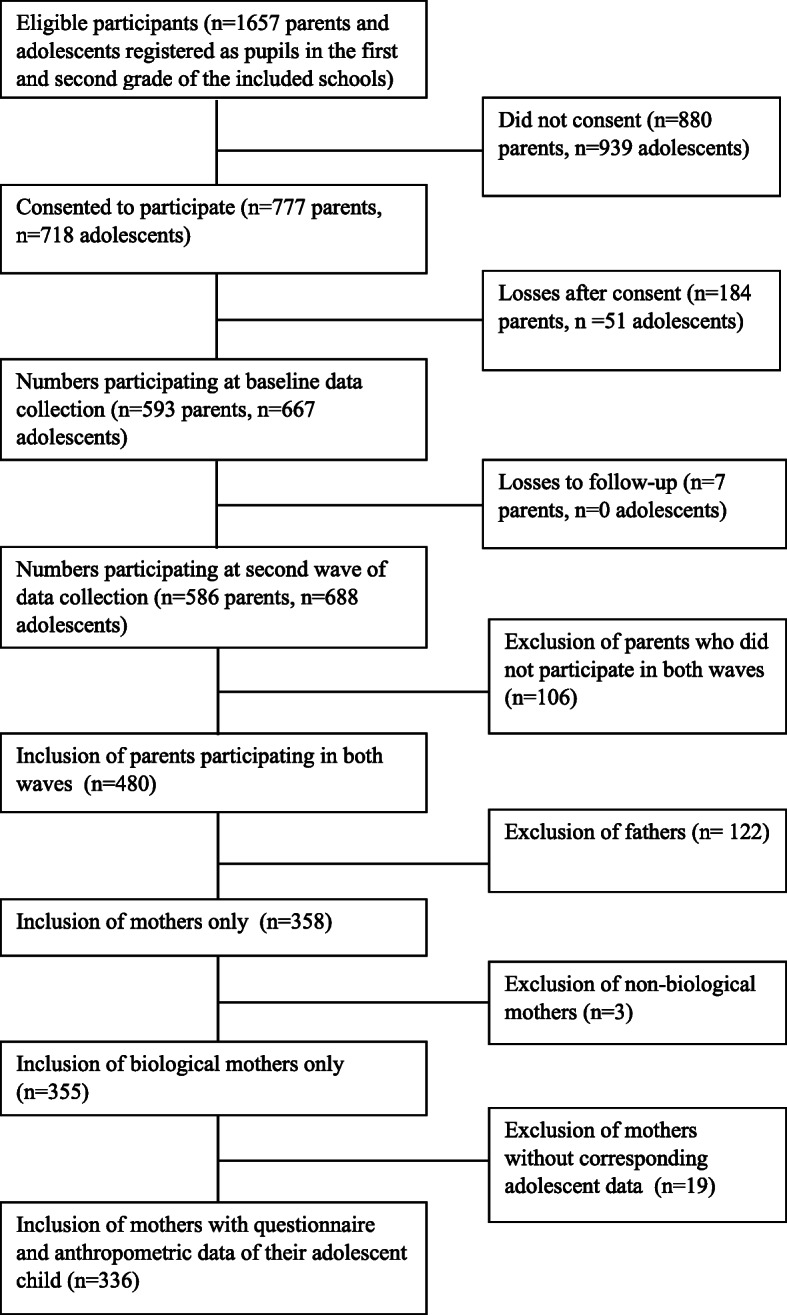
Flow chart of participation in study sample

The participants in the current study were part of a longitudinal study on Dutch adolescents’ and their parents’ health behavior, the “G(F) OOD together” research project. Data for the first three waves were collected in fall 2017, spring 2018, spring 2019 respectively. For this study we used data from the first two waves. Parents of 1657 adolescents were invited to participate, and mothers or fathers provided consent for themselves and their adolescents to participate in the study. Parental consent was provided for 777 parents themselves, of which 593 parents participated in the first wave and for 718 children. Details of the study design can be found elsewhere [71]. For this study, we only included mothers, as they are still the most important caregivers in the family, and are more prone to stress and depression [25, 72, 73] than fathers. In total, 442 mothers took part in Wave 1, 438 in wave 2 (as an extra school was recruited at Wave 2), and 358 (81%) in both waves. Of the *N* = 358 with complete maternal data on both waves, we excluded data from non-biological mothers for this study (*n* = 3) and data from mothers of whom there was no anthropometric and questionnaire data available of their adolescent child (*n* = 19), leaving a final sample of 336 biological mother child dyads who participated in both waves. Figure 1 depicts the participation process in a flow chart [74].

Most mothers (97.0%) were born in the Netherlands. Mean age of mothers at the first wave was 44.6 years (SD_age_ = 4.2; age range = 29.8 to 55.5). Most mothers finished higher professional education (43.3%) or secondary vocational education (36.7%) and performed a payed job of less than 32 h per week (52.5%) or 32 h per week or more (19.4%).

Adolescent boys (*n* = 161) and girls (*n* = 175) were approximately equally represented. Most adolescents were born in the Netherlands (99.1%). All participants attended regular secondary education (M_age_ = 12.9 years; SD_age_ = 0.6; age range = 11.3 to 14.8). In the Netherlands, children in secondary schools follow education based on their academic level and interests. Dutch secondary schools are divided into three streams which represent different educational paths: one to prepare students for vocational training (‘VMBO’), a middle stream to prepare students to study at universities of applied sciences that focus on the practical application of arts and sciences (‘HAVO’) and another to prepare students for university (‘VWO’). More than half of the participants (57.6%) were in pre-university education (‘VWO’), 8.2% of the participants was in higher general secondary education to prepare for applied sciences (‘HAVO’), and 34.2% of the participants was in pre-vocational education (‘VMBO’).

### Procedures

Adolescents and their parents were recruited through secondary schools. We randomly invited 40 secondary schools in the South and the East of the Netherlands to participate in the study. Six secondary schools agreed to participate in wave 1, and all adolescents attending the first and second grade and their parents were invited to participate in this study by means of an active parental consent procedure. Further details on the study procedures can be found elsewhere [71]. A letter describing the four-wave study was mailed to both parents and they were asked to return a (paper or online) consent form indicating whether they agreed to their child participating in the study and if they agreed to participate in the study themselves. If at least one of their parents’ forms was returned, children were rewarded with a small incentive, whether permission was given or not. Before participation, adolescents and parents were informed that participation was voluntary, that they could withdraw from the study at any time and that their answers would be processed anonymously. Inclusion criteria for participants were being enrolled in a high school, being in the first and second grade of this high school, being proficient in the Dutch language and parents and children both having given active informed consent. Exclusion criteria for participants were not being proficient in the Dutch language, attending special education and not having given active (parental) consent.

During one classroom hour of approximately 45 min, adolescents completed an online survey at school, and height and weight were measured outside the classroom by trained research assistants. Parents completed an online survey, which took approximately 20 min to complete. The questionnaires were administered through Qualtrics Survey Software (Qualtrics, Provo, UT, USA) and were in Dutch language. Children received a small present after completing the survey, and several prizes were raffled among participating parents. The Institutional Review Board of the Faculty of Social Sciences of the Radboud University, Nijmegen, The Netherlands approved the study protocol (reference number ECSW20170805–516) in 2017.

### Measures

#### Depressive symptoms

Maternal depressive symptoms were assessed with the 10-item short version of the Center for Epidemiological Studies-Depression (CES-D) scale. The CES-D is widely used and has adequate internal reliability [75]. Respondents rated items on a 4-point Likert scale (rarely or none, to most or all the time). The scale includes positive (I was happy) and negative (I could not get going) items. Higher total CES-D scores reflect greater maternal depressive symptomology. In the current study, Cronbach’s alpha for the CES-D was .77 at T1 and .80 at T2. The test-retest reliability between T1 and T2 (r = .60) was acceptable, taking into account the period of 6 months between the two questionnaires.

#### General perceived stress

Maternal general stress levels were assessed using the 4 item Perceived Stress Scale (PSS). The PSS is a self-report questionnaire measuring a person’s evaluation of stressful situations in the previous 1 month of his or her life. It is a global measure of stress that is simple to use, and there are many studies confirming its reliability and validity in a variety of settings and in multiple languages [76–81]. The instrument contains 4 statements which measure how unpredictable and uncontrollable respondents feel their lives are, for example: In the last month, how often have you felt confident about your ability to handle your personal problems? Respondents rate how often they experience stressful situations on a 5-point Likert scale ranging from ‘never’ to ‘very often’. Answers of the 4 items were summed into a total PSS score. The higher the score on the PSS, the greater the respondent perceives that their demands exceed their ability to cope. Cronbach’s alpha was calculated to investigate the internal reliability of the Perceived Stress Scale and was .70 at T1 and .69 at T2. The test-retest reliability between T1 and T2 (r = .60) was acceptable, taking into account the period of 6 months between the two questionnaires.

#### Financial stress, stress at work and at home

Three types of domain-specific stress were measured through three items: financial stress (How often did you experience financial stress in the past year?), stress at work (How often have you felt stress at work in the past year?), and stress at home (How often did you experience stress at home in the past year?). These questions have been used in previous studies to measure different types of stress and are worded in the same manner [82, 83]. Respondents rated how often they experienced stress in the different contexts on a 4-point Likert scale (never, sometimes, regularly, all of the time). The test-retest reliability for financial stress between T1 and T2 (r = .70) was good, taking into account the period of 6 months between the two questionnaires. For stress at home (r = .60) and stress at work (r = .62) the test-retest reliability was acceptable.

#### Anthropometrics

Adolescents’ height and weight were measured according to protocol [84] by trained research assistants. Body Mass Index (BMI) was calculated as weight in kilograms divided by height in meters squared. Individual age and gender-specific BMI standard deviation scores (z-scores) were calculated using a Dutch representative sample of 0–21-year olds [85, 86]. Mothers reported their own height and weight based on which we calculated maternal BMI.

#### Covariates

We controlled for parent’s and adolescents’ educational level in our regression analyses, as a higher BMI seems to be more frequent in lower educated youth and in youth with lower educated parents [59–61]. We also controlled for additional covariates showing a potential link with adolescent zBMI [62–68]. For example, children are more likely to be overweight or obese in the single-parent (single-mother) family structure [63, 67]. It is known that single household status is associated with having higher time demands due to the lack of shared household responsibilities. Subsequently, a reduction of homemade meals, shared family meals, and physical activity can occur [63, 67]. Moreover, stress perceived by children themselves seems to alter their energy intake and food selection with a preference for sweet and high fat foods [69] and is associated with adolescent weight status [66]. Furthermore, children and adolescents whose mothers are overweight or obese are more likely to be overweight [62, 67, 70], and gender of the adolescent seems to be a risk factor for developing overweight in adolescence [64]. Depressive symptoms experienced by adolescents themselves [64, 65, 87] and a poor quality of the child parent-relationship [68] also seem to be risk factors for overweight in children. As such, the following covariates were added: parent’s and adolescents’ educational level, adolescents’ gender, maternal BMI, maternal single household status, adolescent stress and depressive symptoms, as also the quality of the parent-child relationship rated by parents.

Adolescents’ educational level was coded as 1 = pre-vocational education (‘VMBO’), 2 = higher general secondary education (‘HAVO’), and 3 = pre-university education (‘VWO’). Parents educational level was coded as 1 = primary/high school, 2 = secondary vocational education, 3 = higher professional education/university, following the guidelines of Statistics Netherlands (CBS) [88]. Adolescents’ gender was coded as males = 1 and females = 2. Maternal BMI was calculated as weight in kilograms divided by height in meters squared. Maternal single household status was coded as 0 = married or other significant relationship and 1 = single or no significant relationship. Adolescent stress was measured by asking adolescents to indicate how often they experienced stress at home and at school in the past year on a four point likert scale (rarely or never to most or all the time). We used the mean of these two items by summing them and dividing this by two. Adolescent depressive symptoms were measured with the 10-item short version of the Center for Epidemiological Studies-Depression (CES-D) scale, filled out by adolescents. Parents rated the quality of the parent-child relationship on a slider scale from 1 to 100, 1 being very low quality and 100 indicating a very high quality.

### Statistical analyses

Statistical analyses were conducted using the PASW 20.0 and R software package. Descriptive statistics were used (mean, standard deviations and percentages) to describe the study sample and to investigate population characteristics (see Table 1).

**Table 1:** Sociodemographic characteristics of the study sample

	Total study sample (***N*** = 336 parents and adolescents)
Age of the child (years) at T1; mean (SD)	12.9 (.6)
Range	11.3–14.8
Gender (% male)	47.9
Age of the mother (years); mean (SD)	44.6 (4.2)
Range	29.8–55.5
Educational level mother (%)	
Primary school/high school	9.4
Secondary vocational education (MBO)	36.7
Higher professional education (HBO)	43.3
University	10.6
Educational level adolescent (%)	
pre-vocational education (VMBO)	34.2
higher general secondary education (HAVO)	8.2
pre-university education (VWO)	57.6
Adolescent zBMI at T1; mean (SD)	.10 (1.1)
Range	−2.9 – 2.9
Adolescent zBMI at T2; mean (SD)	.18 (1.1)
Range	−2.8 – 2.9
zBMI categories at T1 (%)	
< −2	1.9
-2 to 2	93.7
2+	4.4
zBMI categories at T2 (%)	
< −2	2.4
-2 to 2	91.7
2+	5.8
Maternal BMI score; mean (SD)	24.6 (4.2)
Range	16.6–43.0
Maternal CESD score; mean (SD)	16.6 (3.1)
Range	10–30
Maternal PSS Score; mean (SD)	7.9 (2.2)
Range	4–18
Maternal financial stress; mean (SD)	1.5 (.7)
Range	1–4
Maternal stress at home; mean (SD)	2.0 (.6)
Range	1–4
Maternal stress at work; mean (SD)	2.1 (.7)
Range	1–4
Marital status (single household status) (%)	10.1
The quality of parent/child relationship as rated by parents; mean (SD)	89.2 (11.6)
Range	17–100
Child stress; mean (SD)	1.9 (.6)
Range	1–4
Child depressive symptoms	14.7 (3.9)
Range	10–33

First, cross-sectional associations between mothers’ wellbeing and adolescent zBMI and the covariates were examined by calculating Pearson’s correlation coefficients using SPSS. Second, to test whether maternal stress or depressive symptoms may precede child zBMI over time, multiple linear regression analyses were performed using the R software package (R Core Team, 2018) with depressive symptoms (CES-D score), general stress (PSS score), financial stress, stress at home or stress at work at T1 as the independent variables, and adolescents’ zBMI at T2 as the dependent variable. We examined two models: a multiple linear regression model, adjusted for adolescents’ zBMI at baseline and potentially relevant covariates (i.e., educational level of the mother and of the adolescent, gender, maternal BMI, maternal marital status (single household status), the quality of parent/child relationship as rated by parents, child stress, and child depressive symptoms and an unadjusted model without covariates but adjusted for zBMI at baseline. We checked normality and distribution assumptions of zBMI before performing our regression analysis with a scatter plot, QQ plot and the Shapiro Wilk test. The plots showed no extreme outliers and a linear association. The Shapiro Wilk test showed a normal distribution (W = .99, *p* = .53).

A logistic regression analysis comparing those adolescents who participated in the current study (score = 1) with those who could not participate because of lacking maternal data (score = 0) showed no differences in gender, educational level, age and mean zBMI between both groups, we therefore expect no bias. The overall proportion of drop-out from T1 to T2 is relatively low, of the adolescents who gave consent, 95.8% participated in both waves and 80.9% of parents participated in both waves. The proportion of missing data in our study sample is also low (22/358 = 6%). To account for missing values we used the ‘na.exclude’ function in R which does not use the missing values, but maintains their position for the residuals and fitted values.

For the proposed multiple linear regressions, we conducted a sample size analysis using G*Power 3.1 [10]. With a small effect size (f2) of .15, an alpha of .05, a standard power level of .80, and a total of 14 predictors, the results of the sample size analysis showed that a minimum of 135 participants would be needed to achieve an appropriate power level for this study. Our study sample of 336 parent-child dyads exceeds this number, indicating sufficient power for the proposed analyses.

## Results

### Cross-sectional associations and descriptives

The total sample consisted of 336 adolescent-parent dyads. Mean zBMI of the adolescents was .10 (SD 1.06) at T1 and .18 (SD 1.07) at T2. The average change in zBMI from T1 to T2 (6 months interval) was .08 (SD .99), as can be seen in Table 1.

Pearson’s correlation coefficients between maternal wellbeing, covariates and adolescent zBMI are presented in Table 2. Significant correlations were found between maternal depressive symptoms and most of the stress measures, with exception of the ‘stress at work’ measure. Mostly non-significant correlations were found between maternal wellbeing (i.e., stress or depressive symptoms) and adolescents’ zBMI. Of the covariates, maternal BMI was positively correlated with maternal general stress (r = .14, *p* = .03), with maternal depressive symptoms (r = .12, *p* = .01) and with financial stress (r = .18, *p* = .001). Educational level of the adolescent was negatively correlated with maternal depressive symptoms (r = −.17, *p* = .003) and with maternal general stress (r = −.15, *p* = .009). Single household status was correlated with financial stress (r = .22, *p* = .000). Quality of parent child relationship was negatively correlated with maternal general stress (r = −.19, p = .001) and stress at home (r = −.22, p = .000).

**Table 2:** Correlational associations, *N* = 336

	1.	2.	3.	4.	5.	6.	7.	8.	9.	10.	11.	12.	13.	14.	15.
**Adolescent variables**															
1. Adolescent zBMI T1	1														
2. Adolescent zBMI T2	.57^a^	1													
**Maternal wellbeing variables**															
3. Maternal depressive symptoms	.01	.02	1												
4. Maternal general stress	.003	.08	.55^a^	1											
5. Maternal stress at work	.000	.000	.10	.17^a^	1										
6. Maternal financial stress	.09	.03	.19^a^	.20^a^	.01	1									
7. Maternal stress at home	.04	−.01	.27^a^	.34^a^	.14^b^	.20^a^	1								
**Covariates**															
8. Gender adolescent	−.001	.04	.01	.003	.02	.03	.06	1							
9. Depressive symptoms adolescent	.07	.08	.06	.01	.03	.10	.10	.15^a^	1						
10. Adolescent stress	.03	−.01	.05	.08	.03	.02	.08	.10	.50^a^	1					
11. Eucational level adolescent	−.15^a^	.03	−.17^a^	−.15^a^	.01	−.09	.01	.05	−.09	−.02	1				
12. Educational level parent	−.10	−.09	−.09	−.07	.18^a^	−.08	.07	.06	.06	.06	.34^a^	1			
13. Maternal BMI T1	.30^a^	.20^a^	.14^b^	.12^b^	−.01	.18^a^	−.04	.01	.04	.02	−.22^a^	−.17^a^	1		
14. Relationship status parent	.02	−.04	−.02	.02	.03	.22^a^	.10	.05	.14^b^	.06	−.08	−.02	−.03	1	
15. Quality of parent/child relationship	−.02	−.05	−.07	−.19^a^	−.08	.02	−.22^a^	.10	−.05	−.03	−.003	−.10	−.004	−.08	1

^a^ significant at the 0.01 level (2-tailed). ^b^ significant at the 0.05 level (2-tailed)

### Longitudinal associations between maternal mental wellbeing and adolescents’ zBMI

Maternal general stress (β = .20, *p* = .002) was associated with adolescent’s’ zBMI at T2 after correction for baseline zBMI, gender, educational level of the child, educational level of mothers, maternal marital status (single household status), the quality of parent/child relationship as rated by parents, child stress, and child depressive symptoms and maternal BMI as can be seen in Table 3. This effect was still significant in a reduced model without covariates (β = .14, *p* = .01), indicating that maternal general stress is associated with higher zBMI 6 months later. Notably, for the other maternal well-being variables (i.e., maternal financial stress, maternal stress at home, maternal stress at work, maternal depressive symptoms) no significant longitudinal associations were found with adolescents’ zBMI, neither in a reduced model nor after controlling for covariates. Additionally, lower educational level among adolescents (β = .16, *p* = .001) and adolescent depressive symptoms (β = .16, *p* = .001) were associated with a higher zBMI at T2. The final model (including all covariates) of maternal general stress explained 31% of the variance of adolescent zBMI.

**Table 3:** Linear regressions of maternal stress/depressive symptoms at T1 on adolescent zBMI at T2

	Analyses adjusted for baseline zBMI ^**a**^				Analyses adjusted for covariates ^**b**^			
	B	SE B	β	***P*** value	B	SE B	β	***P*** value
**Maternal general stress**	.08	.03	.14	.01*	.10	.03	.20	.002**
**Maternal depressive symptoms**	−.01	.02	−.03	.49	−.02	.02	−.05	.44
**Maternal financial stress**	−.05	.08	−.03	.50	−.07	.08	−.04	.41
**Maternal stress at home**	−.07	.09	−.04	.43	−.09	.09	−.06	.30
**Maternal stress at work**	−.00	.08	−.00	.97	.004	.08	.003	.95
**Adj R** ^**2**^	.35				.31			

** significant at the 0.01 level (2-tailed). * significant at the 0.05 level (2-tailed). ^a^ Adjusted for: adolescents’ zBMI at baseline ^b^ Adjusted for covariates:

adolescents’ zBMI at baseline, educational level, gender; maternal BMI, educational level of mothers, maternal marital status (single household status), the quality of parent/child relationship as rated by parents, adolescent stress, and adolescent depressive symptoms

## Discussion

Longitudinal studies that test whether maternal stress or depressive symptoms may precede the development of adolescents’ weight outcomes are rare, and this study aimed to fill this gap. We found that maternal general perceived stress at T1 preceded higher adolescents’ zBMI at T2, after controlling for baseline zBMI and other covariates, whereas maternal depressive symptoms at T1 and other domain-specific stress did not (maternal financial stress, maternal stress at work, maternal stress at home). Additionally, lower educational level among adolescents and adolescent depressive symptoms were associated with a higher zBMI at T2.

In the past decade, several studies have established the link between maternal well-being and childrens’ zBMI [28–31, 33]. However, these studies mostly focused on (early) childhood. During adolescence, particularly early adolescence, parents still play an important role in their children’s lives. Maternal stress and depressive symptoms have been linked to barriers to a healthy lifestyle and may reduce pro-active obesity-related parenting practices [29, 89–91] such as less healthy meal preparation and less transportation to and less participation in organized sports by their children, and by for example, negatively influencing mother-child interaction and increasing the risk at modelling possibilities of unhealthy maternal behaviors [25, 36, 39, 40, 45–51, 91, 92]. The present study’s aim was to investigate the longitudinal link of both maternal stress and depressive symptoms with adolescents’ weight development, and we hypothesized that maternal stress and depressive symptoms are linked with increases in adolescents’ zBMI development over time. We found a small link between maternal general stress and adolescent zBMI over time, but counter to our hypothesis no significant associations for maternal depressive symptoms or for domain specific stressors (maternal financial stress, stress at home and stress at work) were found.

Maternal general perceived stress may reflect a broader personality construct on how mothers more generally react to stressful situations, whereas stress from the home and work environment probably is more contextual in nature [34]. This may explain why maternal general stress is associated with development of zBMI in adolescents, having more general impact than specific contextual stress factors. Additionally, adolescents with mothers who experience a lot of general stress may be exposed to less healthy family and peer environments, providing increased opportunities to engage in unhealthy behaviors and may lead to unhealthy weight development.

In contrast, no associations were found between maternal depressive symptoms and adolescent zBMI over time. Notably, previous research has already shown some mixed findings particularly with regard to the link between maternal depressive symptoms and children’s weight outcomes [54, 55]. A review reports that chronic depression (depression measured on multiple occasions), but not episodic (depression at a single measurement occasion) depression was found to be associated with a greater risk for child overweight [54]. In our study we measured depressive symptoms at one time point, which may explain why we did not find any associations. However, it should be noted that other studies focusing on episodic symptoms did sometimes find a link with children’s weight outcomes [93, 94], though these studies were most often conducted among younger children.

The link between maternal stress and children’s weight outcomes have been repeatedly found among families with younger children [28]. To the best of our knowledge, only one previous longitudinal study found this link among adolescents [33]. Our study adds to these previous studies that diverse stress factors have been examined and that only for one specific stress factor longitudinal links have been found (i.e., general stress) in an adolescent population. Thus, it might be that, particularly for adolescents, maternal general stress has more impact on (healthy) family life and adolescents’ weight development than maternal stress in other domains and also more impact than maternal depressive symptoms. Future studies including both age groups (i.e., younger children and adolescents) may further examine this.

Socioeconomic factors are also known to be of specific interest in weight development of children and adolescents. We found that a lower educational level among adolescents was consistently associated with a higher zBMI at T2 in all our models. In the Netherlands, children in secondary schools follow education based on their academic level and our study thus suggests that increases in zBMI are most unfavorable in adolescents with a lower educational level. Previous studies have reported about the educational gap with regard to weight outcomes [95]. It seems that the period of adolescence is a particularly important period because of the autonomy involved in making more independent choices about weight related behaviors. Adolescents with lower educational levels may make more unhealthy choices in their weight related behaviors than highly educated adolescents, and may have less (financial) opportunities for healthy weight related behaviors.

Our findings considering adolescent depressive symptoms which consistently were associated with a higher zBMI at T2 in our models are in line with findings of longitudinal studies that depressive symptoms, or depression episodes, during childhood or adolescence are linearly associated with increases in body mass index (BMI), overweight, or obesity over time [87, 96–99]. The link between maternal mental health and child weight development through child mental health issues may be particularly important during adolescence, when depression and stress symptoms are more prevalent compared to childhood [53].

The current study had several strengths and limitations. One particular strength is that height and weight of the adolescents were objectively measured. Another strength includes the fact that parents reported on their own mental health. Additionally, diverse forms of mental health issues, such as general stress and stress which was domain-specific as also depressive symptoms were measured. Despite these strengths, some limitations should be acknowledged. First, the refusal rate on a school level may seem rather high (with 6 out of 40 schools participating). However, the following should be taken into consideration; in the Netherlands, secondary schools are often overloaded with requests for participation in research activities, resulting in the fact that schools are very selective about participation and refusal rates are normally high. With regard to refusal rates at the individual level, the rates are average for the situation in our country. We used an active parental consent procedure. It is known that response rates are higher when passive parental consent (opt-out consent) procedures are used, with estimates as high as 90%, compared to 30–60% for active parental consent procedures [100, 101]. Researchers have consistently found that active parental consent procedures result in lower response rates compared to passive parental consent procedures among middle and high schools students [102, 103]. Our participation rate of 43% is quite average in this respect. As a second limitation, maternal mental health was analyzed at one time point only, to assess stability in maternal mental state more time points should be taken into account. Third, the time period between the first two waves was relatively short and may not be representative of longer term weight change developments. Fourth, the sample consisted of a high percentage of highly educated respondents (57.6%) as well as a high proportion of respondents having a healthy zBMI, possibly influencing the generalizability of the results. School samples recruited with active parental consent procedures are known to be less diverse and have fewer high-risk participants [103]. Additionally, domain-specific stress factors (i.e., financial, home or work-related stress) were only measured with one item. Finally, although we based our research question and analyses on a body of literature that examines the links between maternal stress and adolescent BMI, hypotheses on differential links are quite speculative. As such, we did not formulate separate hypotheses for these differential stress variables.

## Conclusions

To conclude, our findings suggest that adolescents whose mothers experienced more general stress may be at greater risk for increases in zBMI. In contrast, maternal depressive symptoms do not predict any changes in weight development among adolescents. Interventions to increase resources for mothers to cope with stress might help reduce overweight and obesity in their adolescent children. As an additional strategy to address adolescent obesity prevention and intervention, behavioral interventions to help reduce stress such as mindfulness-based stress reduction, could be considered to minimize the effects of stress in mothers on their children’s weight-related behaviors. Our findings warrant future longitudinal investigation with longer follow-ups on whether and how general stress among mothers might predict weight increases of their adolescent offspring.

## Data Availability

The datasets generated and analysed during the current study are not publicly available due to agreements we have made concerning the exchange and use of our data, but are available from the corresponding author [MK] on reasonable request. These data are primary data acquired by (one of) the authors.

## Abbreviations

BMI: Body Mass Index
CES-D: Center for Epidemiological Studies-Depression scale
PSS: Perceived Stress Scale
VMBO: The VMBO is a four-year vocationally-orientated stream focussed on practical knowledge, which leads to vocational training (MBO). It has two qualification levels and students complete the track at the age of 16
HAVO: The HAVO is a five-year middle stream that prepares students to study higher professional education at universities of applied sciences, where they can follow a bachelor’s degree in applied sciences (HBO). Students complete the HAVO around the age of 17
VWO: The VWO is a six-year education stream with a focus on theoretical knowledge, that prepares students for a research university (WO). Students complete the stream around the age of 18
